# An ethics safe harbor for international genomics research?

**DOI:** 10.1186/gm503

**Published:** 2013-11-22

**Authors:** Edward S Dove, Bartha M Knoppers, Ma’n H Zawati

**Affiliations:** 1 Centre of Genomics and Policy, McGill University, 740 Dr. Penfield Avenue, Suite 5200, Montreal H3A 0G1, Canada

## Abstract

**Background:**

Genomics research is becoming increasingly globally connected and collaborative, contesting traditional ethical and legal boundaries between global and local research practice. As well, global data-driven genomics research holds great promise for health discoveries. Yet, paradoxically, current research ethics review systems around the world challenge potential improvements in human health from such research and thus undermine respect for research participants. Case reports illustrate that the current system is costly, fragmented, inefficient, inadequate, and inconsistent. There is an urgent need to improve the governance system of ethics review to enable secure and seamless genomic and clinical data sharing across jurisdictions.

**Discussion:**

Building on the international privacy 'safe harbor’ model that was developed following the adoption of the European Privacy Directive, we propose an international infrastructure. The goal is to create a streamlined and harmonized ethics governance system for international, data-driven genomics research projects. The proposed 'Safe Harbor Framework for International Ethics Equivalency’ would consist in part of an agency supporting an International Federation for Ethics Review (IFER), formed by a voluntary agreement among countries, granting agencies, philanthropies, institutions, and healthcare, patient advocacy, and research organizations. IFER would be both a central ethics review body and also a forum for review and follow-up of policies concerning ethics norms for international genomics research projects. It would be built on five principle elements: (1) registration; (2) compliance review; (3) recognition; (4) monitoring and enforcement; and (5) public participation.

**Summary:**

A Safe Harbor Framework for International Ethics Equivalency would create many benefits for researchers, countries, and the general public, and may eventually have application beyond genomics to other areas of biomedical research that increasingly engage in secondary use of data and present only negligible risks. Among the benefits, research participants and patients would have uniform adequate protection, while researchers would be ensured expert ethics review with a reduction in cost, time, administrative hassle, and redundant regulatory hurdles. Most importantly, society would enjoy the maximization of the potential benefits of genomics research.

## Background

Has biomedical research progressed beyond the individual, the institutional, and the national to one of international collaboration between research groups [1]? While it may be too soon to declare that we have definitively entered a new age, it certainly rings true that the scale and intensity of biomedical research connectivity, especially in genomics and enabled by bioinformatics and cloud computing, has now reached extraordinary levels. This fact challenges the traditional ethical and legal boundaries between global and local research practice [2-6].

Indeed, there has been incredible growth in biobanks, genetic databases, and large genomics research consortia spanning multiple jurisdictions, such as the 1000 Genomes Project [7], the Human Heredity and Health in Africa (H3Africa) Initiative [8], the International Cancer Genome Consortium [9], and the International Rare Diseases Research Consortium [10]. An ever-growing volume of de-identified genomic sequence data and clinical data is being deposited into shared research databases such as the eMERGE Network [11], dbGaP [12], the European Bioinformatics Institute (EBI) [13], and the DNA Data Bank of Japan [14]. Regional and international organizations are working to foster broader genomics research collaboration, as seen in the recent establishment of a European Research Infrastructure Consortium for the Biobanking and Biomolecular Resources Research Infrastructure (BBMRI) [15] and the Global Alliance for sharing genomic and clinical data [16]. Regulatory agencies are also supporting broader and more international collaboration, as seen in the U.S. National Institutes of Health’s (NIH) proposed genomic data sharing policy [17], the European Medicines Agency’s current development of a policy on the proactive publication of clinical trial data [18], and the U.S. Food and Drug Administration’s proposal to make publicly available de-identified and masked non-summary safety and efficacy data derived from medical product applications [19].

Both inside and outside the health research context, data now flow unconstrained in all directions. Though this landscape of internationally collaborative data-driven research is championed by many for holding great promise to accelerate health discoveries and applications, the current underlying research ethics review system seriously challenges the realization of this potential. Indeed, the system encumbers improvements in human health that could accrue from global collaboration, and paradoxically may not improve respect for persons who participate in research [20,21].

In most countries, research involving identifiable individuals or information requires the informed consent of research participants and research ethics committee (REC) approval at each project site. RECs function to safeguard the dignity, rights, safety, and wellbeing of actual or potential research participants [22,23]. This role remains primary, but the current structure of site-specific REC review disproportionately burdens projects that pose only negligible risks to participants and makes little sense in an era marked by the massive aggregation and analysis of data.

Today, the nature of health research is vastly more varied than the classic, physically risky specific disease studies on 'human subjects’ that gave rise to the ethics codes of the mid-to-late 20th century [24-26]. It is also fundamentally different. Researchers inductively 'trawl’ through data to find patterns [27], but also engage in massive aggregation and analysis of data and samples that were initially collected for one disease and are now being used to study another [28]. This includes consolidating prospective or retrospective population cohorts and pooling datasets, such as current and legacy collections of health, lifestyle, and environmental data, to facilitate international, large-scale, collaborative, longitudinal, or remote analyses of samples to better understand complex disease etiology [29].

Numerous case reports illustrate that the current system for ethics review of multi-site research projects, particularly with respect to cross-organizational collaboration, is costly, fragmented, inefficient, inadequate, and inconsistent [30-33]. A UK study from 2006 found, for example, that the overall level of agreement regarding 18 protocols among three different RECs was only slightly better than chance [34]. And a recent qualitative study of 46 investigator’s experiences of RECs in the U.S. (termed institutional review boards, or IRBs) noted that: 'Most investigators expressed concerns that differing views of risk as well as logistical variations across IRBs discouraged multi-site research at a time when large samples are needed to advance science’ [35].

Simply put, the current ethics review governance system in much of the world is not designed to balance patient and participant protection with promising genomics research because the nature of that research has fundamentally changed. We are now in an age of constant transborder data flows and secondary use research of all kinds of data. Yet, the system that evaluates that research for ethical validity remains siloed, territorial, and wedded to a 'classic model of one research scientist or team working in a single lab or clinic and attempting to determine the effectiveness of a new drug or device’ [36]. Research projects that transcend political boundaries and thrive on borderless but secure sharing of data and knowledge invariably must confront the contested boundaries of research ethics review. Must the scientific and medical community still rely on multiple REC reviews for international data-driven projects when these reviews are often inconsistent and unduly dilatory? Are the rights of patients and participants safeguarded if they donate data to contribute to greater improvements in health discoveries, yet later discover that the projects have not yet proceeded or cannot proceed on 'ethical’ grounds?

This latter observation causes the most concern regarding the current system. There are serious practical implications of an ethics review process stuck in a 20th-century paradigm that is single site-specific and is designed for potentially physically harmful interventional clinical trials. For genomics researchers working in international data-driven projects, overlapping and often contradictory multi-site ethics review can hinder their ability to work with globally and socially distributed data and to make research discoveries that improve health. Everyone is consequently affected, from researchers to patients to the broader public.

Proposed reforms at national levels [37-41] are an improvement and particularly appropriate in a non-interventional, data-driven research context. However, by still relying on comprehensive ethics review at each project site in each country, the potential for global bottlenecks and incongruence remain, with multiple RECs reviewing a research project that seeks to aggregate and use data on a global scale.

It is time to shift the paradigm. In furtherance of the vision and values of the ELSI 2.0 Initiative that seeks to develop new frameworks to overcome current barriers to international, interdisciplinary research [42], we call on stakeholders to critically evaluate the current ethics review system and consider what kinds of reform may be desirable and feasible. To this end, we propose a federated 'Safe Harbor Framework for International Ethics Equivalency’ (Safe Harbor) that facilitates the harmonization of ethics review of data-driven international genomics research projects while respecting globally transposable research ethics norms and principles.

## Discussion

### The safe harbor framework

We define a safe harbor as a process, system, or framework that allows a bona fide entity to perform certain actions in compliance with defined standards or conditions in exchange for protection from ordinary regulatory burdens [43]. Safe harbors have been implemented in numerous areas of the law. They carry particular resonance in privacy and health regulation [44,45]. One of the most well-known safe harbors is the U.S.-EU Safe Harbor Framework agreed to in 2000 that allows American companies to receive personal data from EU countries without violating the 1995 EU Data Protection Directive [46]. Our proposal seeks to build on this model by scaling it above the regional level (between Europe and the U.S.) to the international level. The principle behind the safe harbor is the same, regardless of its geographic scope. What brings multiple jurisdictions together in building a safe harbor is the recognition that different regulatory and political systems do not equate to incompatible values, especially in the protection and advancement of human wellbeing and the promotion of biomedical research.

**Figure 1:**
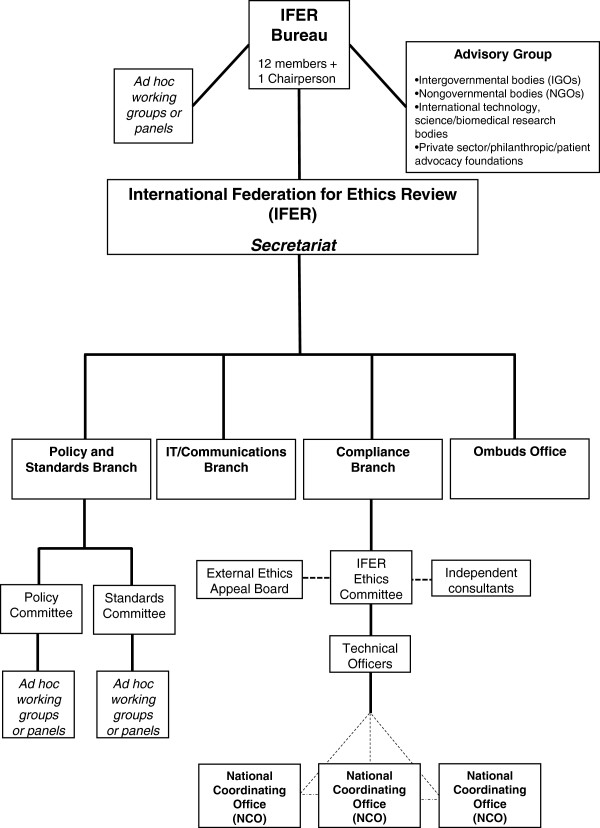
**Organization chart of the Safe Harbor’s primary component, an International Federation for Ethics Review (IFER).** IFER would be constituted by a voluntary agreement among countries, granting agencies, philanthropies, institutions, and healthcare, patient advocacy, and research organizations. The dotted lines in the figure represent *ad hoc* or external parts of IFER. In particular, the IFER Ethics Committee can call upon a standing list of independent consultants who could provide special expertise to the Committee on proposed research projects, be it in methodology, disease, or legal domain. Applicants whose first appeal is rejected by the IFER Ethics Committee may further appeal to the External Ethics Appeal Board. Additionally, NCOs are a key feature of the Safe Harbor but are external to IFER; they work with the technical officers and the Compliance Branch, and coordinate with each other for each research project, but are situated in their own country and are subject to their country’s laws and regulations.

As depicted in Figure 1, our Safe Harbor would consist of an international agency built on five principle elements: (1) registration; (2) compliance review; (3) recognition; (4) monitoring and enforcement; and (5) public participation. The agency’s mission would be to connect governments around the world to harmonize where possible ethics review guidelines and policies, increase ethical conduct, and ensure compliance for researchers involved in a clearly defined type of genomics research project (Box 1), all within a nimble and agile system supported by substantive principles (Box 2) and procedural mechanisms (Box 3).

In recognition of the longstanding work occurring in related fields, clinical trials with pharmaceutical products or devices would remain excluded from the Safe Harbor and should remain subsumed within the International Conference on Harmonisation of Technical Requirements for Registration of Pharmaceuticals for Human Use (ICH) framework [47].

Furthermore, while in the long run we envision the Safe Harbor having the authority to handle a broad array of international research projects, we believe that in the short term, the greatest chance of success necessitates a focus on just one critical area of data-driven research: genomics. As legal scholars and scientists recently noted in a study on the proposed revisions to the U.S. Common Rule in the context of evolving large-scale research projects like the Human Microbiome Project (HMB), 'While a change in the Common Rule to streamline IRB approval of multisite studies or mandate a single IRB for multisite studies would be a benefit to the HMP and other similar “big science” research studies, it may make more sense to consider the type of research being proposed rather than to mandate this change for all multisite research studies’ [36]. We agree with this sentiment, but add that depending on its feasibility and viability, we hope that the Safe Harbor’s scope could later be expanded.

### International federation for ethics review

Harmonizing ethics review for international data-driven research projects requires international ethics governance reform. An individual country may work towards reducing redundancies in ethics review and aim to create efficiencies for multi-site studies, but usually such reform stops at the political boundary. National reform alone does not and cannot address international concerns. Policymakers, researchers, and other stakeholders who wish to remedy the systemic problems in ethics review could support an international organization that is capable of steering globally collaborative research projects to an ethical safe harbor. The chief component of the proposed Safe Harbor, therefore, is a newly constituted organization. In line with the goals of the Global Alliance [16], which promotes the responsible sharing of genomic and clinical data and international interoperability and harmonization, we suggest an IFER formed by a voluntary agreement among countries, granting agencies, philanthropies, institutions, and healthcare, patient advocacy, and research organizations.

As constituted by a foundational Charter and governed by internal Rules of Procedure, IFER would be both a central ethics review body engaged in deliberation of the possibly divergent ethical aims of funders, institutions, research organizations, and participants, and also a forum for review and follow-up of policies concerning ethics norms for international research projects. IFER’s budget could be established and maintained by requiring research projects or their funding agencies to pay for the registration submission and ethics review, and collecting dues from member countries based on their ability to pay. As depicted in Figure 1, the Agency would be comprised of several parts. A Bureau would serve as the executive arm and consist of a Chairperson and a multidisciplinary panel of independent experts. Assisting the Bureau with its core functions would be an Advisory Group comprised of multiple types of organizations that would keep IFER abreast of the changing realities and needs of technology and data, as well as laws, regulations, and policies governing ethics review and human subjects research.

IFER would have four internal branches, with staff members appointed by the Board. An Ombuds Office would receive, investigate, and address complaints of both internal IFER concerns and alleged research project ethics violations; it would report its findings and recommendations for changes to policies or procedures to the IFER Bureau. A Policy and Standards Branch would create, revise, and interpret policies and standards that govern the ethics review process and related ethical issues, such as confidentiality, consent, and conflicts of interest. Within this branch, the Policy Committee would be charged with the policy component of IFER, while the Standards Committee would be charged with developing standards for operationalizing IFER’s policies. An IT/Communications Branch would maintain the IFER website and access portals; it would also coordinate ethics educational and factual information dissemination and communication flows between researchers, National Coordinating Offices (discussed below), and the public. Finally, a Compliance Branch would manage the ethics review of research projects and ensure ongoing and prospective compliance with the IFER-promulgated policies and standards.

### Registration and national coordinating offices

**Figure 2:**
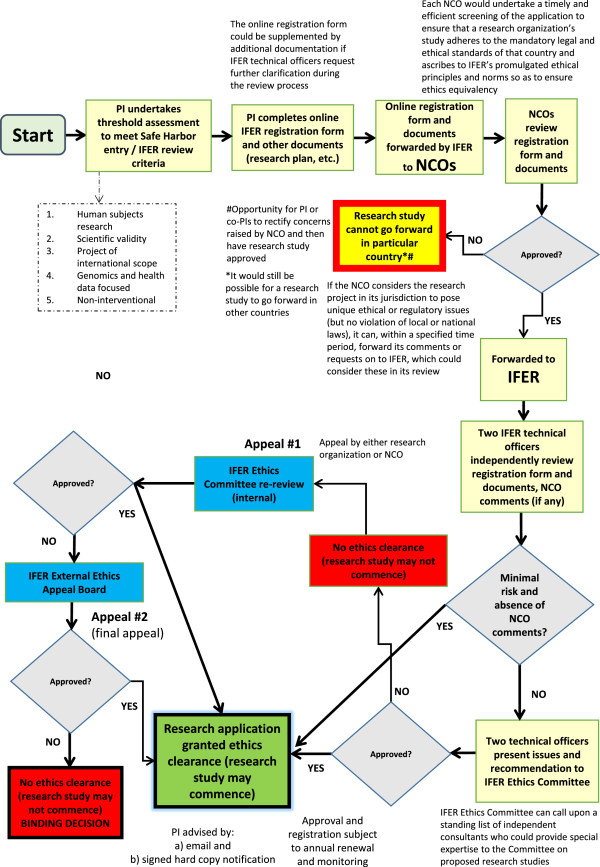
**Safe Harbor flow chart.** Interested applicants who are undertaking an international, multi-site data-driven genomics project would be able to partake in the Safe Harbor Framework for International Ethics Equivalency, whose ethics review mechanism is represented in this flow chart. The process includes the PI(s) completing an online IFER registration form and other relevant documents (research plan, and so on), undergoing streamlined NCO screening and IFER review, and having the opportunity to appeal a decision.

As depicted in the flow chart in Figure 2, the type of research project determines its inclusion in the Safe Harbor. By participating in the Safe Harbor, healthcare, research, and disease advocacy organizations that plan to conduct an international, multi-site data-driven genomics project would avoid multiple REC review within and across countries but still satisfy the local context concerns of the countries wherein the project is based. They must meet specified criteria in a publicly available and standardized online form that requires several disclosures, including a research plan that conforms to a recommended format and describes the anticipated research procedures, benefits, risks, and burdens.

Before IFER’s technical officers undertake streamlined ethics review, each country and institution that hosts a site (or sites) in a research project would have a vital role to play. Indeed, the formation of an international agency tasked with ethics oversight is impossible without the explicit buy-in of governmental bodies and institutions. Each country’s governmental agency (and state or provincial equivalent) that is responsible for human subjects ethics review oversight would sign onto IFER via a revocable, voluntary agreement. Additionally, institutions could sign onto IFER. The agreement between IFER and a country would require the country to abide by an IFER Charter and Code of Conduct and to create a National Coordinating Office (NCO) for the specific types of research projects appertaining to the Safe Harbor. The agreement would mandate IFER to distribute the online registration form and additional documentation (for example, research plan, consent form) to the NCOs where a research project is planned.

As the emphasis in the Safe Harbor is on streamlined and efficient ethics review, including due consideration to conditions that reflect unique circumstances on the ground, each NCO would carry out a timely and efficient screening of the application to ensure that a research project adheres to the mandatory legal and ethical standards of that country and, if applicable, state or province through communication with local agencies that are charged with human subjects research. These standards could range from laws and policies on human rights, privacy or data protection, to research involving humans or human biological materials. An NCO could inform IFER upon screening that a project cannot go forward in its country because it violates local or national guidelines or laws, though the goal of IFER, in promulgating ethical best practices and interoperability for international genomics research projects, would be to minimize such a determination. While some may view the NCO as just another REC, in fact it streamlines the ethical review process. An NCO reduces the regulatory burden for international genomics projects as there would be coordinated and streamlined screening at one central location in a country as opposed to multiple, overlapping ethics reviews at locations scattered throughout a country, often at significant cost, delay, and uncertainty to researchers.

While one NCO per country vastly improves the multiple-REC-per-country situation, problems could still arise. In particular, because not all NCOs may be alike in the rigor they apply to application screenings, IFER’s Compliance Branch should periodically audit NCO determinations to assess their consistency across time and their variation among other NCOs. The Compliance Branch should also monitor the potential for any adverse 'forum shopping’ that could arise where applicants design their projects to take advantage of NCOs that are viewed as considerably less stringent in their screening process, or to bypass IFER review altogether by submitting applications to local RECs. Further compliance review by IFER’s technical officers should assuage some of these concerns, but during the initial online registration stage, applicants should be required to disclose whether they have previously submitted their research proposal to any local or regional RECs or NCOs, and if so, to disclose which RECs or NCOs and the outcome of the reviews.

### Compliance review

Once each NCO undertakes its preliminary screening, and assuming the NCO determines that the research project adheres to mandatory legal and ethical standards, it would then send its approval letter or comments on to IFER via a secure online portal. Two IFER technical officers would then confidentially review the application material and compare it to publicly available ethical norms and procedural safeguards established by IFER’s Policy and Standards Branch that promote internationally consistent and substantially equivalent ethical assessment of large-scale data-driven genomics research projects. The non-interventional, minimal risk nature of research projects within IFER calls for a stronger deliberative platform for researchers and higher thresholds to require changes to a research project design [48]. If both technical officers independently determine that the submitted application presents only minimal risk to research participants, and no NCO has submitted ethical issues of concern that require full IFER Ethics Committee deliberation, the application would be approved and exempted from further IFER review. Minor issues of concern raised by one or both technical officers should be resolved through negotiation with the principal investigator (PI) and/or co-PIs.

Applications with NCO comments of concern attached or that present more than minimal risk would be forwarded for review by a committee of technical officers (IFER Ethics Committee) who have special expertise in the proposed research project. At this stage, the PI should be provided the opportunity to present the proposal or elaborate on specific issues in person or via web conferencing. Following a general discussion, the Committee would then make a consensus decision in a timely manner (approved as submitted; conditional approval; deferred decision; or not approved) that reflects an ethical judgment about the permissibility of the research project. As Figure 2 depicts, concerns of concentrated power are assuaged with a two-step appeals process for review decisions.

### Recognition

Information technology would drive the Safe Harbor, ensuring adequate review, communication, oversight, and public participation. The IFER website, coordinated by the IT/Communications Branch, would contain separate portals for the public, international consortia, and governments, each with FAQs and additional information for educational purposes. It would maintain a current, up-to-date registry and digital archive of IFER-approved research projects, along with a lay summary of each project, the rationale for IFER’s approval of a project so that other researchers may learn how to design an ethically valid project and to promote continuous quality improvement, and site-specific contact information should participants or members of the public wish to obtain further information.

### Monitoring and enforcement

The Safe Harbor requires a strong regulatory system that can receive and investigate complaints, monitor and evaluate compliance, ensure enforcement, and promote an international Code of Conduct for clear and consistent behavior by all actors within a well-defined scope. A Code of Conduct should include a PI’s obligation to provide IFER’s Compliance Branch with current information about the project and an agreement to be subject to occasional audits or ongoing assessments. It should also include mandatory notification by the PI to IFER and all applicable NCOs upon a data breach or other defined ethical lapse, strict enforcement and penalties for data breaches or serious ethical lapses, and a prohibition on attempted individual re-identification if personal data is coded or anonymized. An independently functioning Ombuds Office would receive, investigate, and address complaints of possible ethics violations and report its findings and recommendations for changes to policies or procedures to the IFER Bureau. The Ombuds Office would also liaise with NCOs, who could investigate and address complaints at local project sites, and pass along information to a research project’s funders or institutional administrators.

### Public participation

Public accountability and trust in a regulatory system are best cultivated in an environment of participation and transparency [49]. IFER should, in addition to being transparent about its decisions (both for proposals approved and not approved) and the rationales for those decisions through annual reports and online disclosures, seek a plurality of views. This could prevent ethical and governance blind spots and encourage the evaluation of current ethical norms and consideration of possible new ones. Resources and interest permitting, IFER could hold annual or biannual live-streaming conferences at the Secretariat that are open to all members of the public. The conferences could serve as a forum to review policies and standards and assess recent work done by IFER, including a review of Ethics Committee decisions. All participants would have opportunities to comment and possibly even vote on the review or adoption of new policies. By having publics come together and deliberate, the IFER Bureau and staff would be encouraged to continually reassess the Safe Harbor and scrutinize it in light of new information and diverse perspectives.

There are practical challenges to such transparency, of course. Holding annual or biannual conferences at the Secretariat to improve transparency and public participation, and handling the multiple languages that are likely to be spoken by publics attending the conferences, are difficult. However, this is not particular to the Safe Harbor Framework, as international conferences almost always present the same practical challenges, and quite often can be addressed by diligent planning, including the provision of competent interpreters and state-of-the-art technology. The European Commission’s Directorate General for Interpretation [50], which serves as the interpreting service and conference organizer for the European Commission, is a prime example of how an international organization can work towards giving every participant a voice in their own language with minimal obstacles to understanding.

## Summary

While our proposal is only briefly sketched here, it will likely raise objections, particularly with respect to cost, political buy-in, local context, and regulatory complexity. To the latter objection, we suggest that in a world of Big Science and Big Data, Big Ethics is needed. Policy complexity and some bureaucracy are inevitable when attempting to comprehensively remedy as complex a governance issue as the research ethics review system. Though a Big Ethics structure like the Safe Harbor may be criticized for complexity, it is drastically less than the bewildering complexity caused by multi-site ethics review fragmentation experienced around the world currently.

Moreover, we think the Safe Harbor is both a desirable and feasible proposal because the fragmented status quo research ethics review structure is increasingly losing functionality and legitimacy. As more international genomics projects are launched around the world, particularly in developing regions, researchers, participants, and patients alike will experience the adverse effect of delays and deficiencies in the ethics review process. A rising tide of criticism by scholars and researchers directed towards funders and regulatory agencies will encourage a move towards governance reform to some degree. The only question is to what degree. We propose fundamental reform, and one that will involve minimal effort on any one country’s part. This is the greatest benefit of an international agency. Like the genomics research projects it reviews, the Safe Harbor is an internationally collaborative endeavor that catalyzes the strengths of each participant country.

Our proposal offers a novel structure, but the purpose and rationale is linked to precedents in other areas of global biomedical research where international collaboration has successfully improved underlying governance and regulatory structures. For example, the ICH was founded in 1990 and is comprised of both regulatory bodies and research-based industry in the EU, U.S., and Japan. Its Global Cooperation Group is widely acclaimed for achieving international harmonization in the interpretation and application of technical guidelines and requirements for pharmaceutical product registration. This has reduced duplication of testing carried out during the research and development of new drugs and has spread the realization of global health worldwide [47].

Likewise, the benefits of a Safe Harbor Framework for international ethics review harmonization could be far-reaching. Researchers would enjoy ensured expertise on the research under review, as well as a significant reduction in cost, time, administrative hassle, and redundant regulatory hurdles. An international Code of Conduct that requires NCOs or designated agencies with statutory authority to dole out strict sanctions for violations of the Safe Harbor principles and standards ensures research participants and citizens that a streamlined ethics review process does not mean a reduction in oversight or enforcement. To the contrary, it means more efficient review and increased monitoring and sanctioning for ethical or legal transgressions. Countries can also benefit from the Safe Harbor since the consolidation of ethics review into the NCO would allow their genomics research sector (and later, one hopes, the broader biomedical research sector) to save money and time otherwise spent on multi-site and often contradictory REC review. Countries will add value to their society through improvements in healthcare and public health, which has individual benefits but also collective economic benefits by way of increased economic development and productivity [51].

Undue burdens are borne by researchers, research participants, and society in general because of the current ethics review system. A Safe Harbor Framework for International Ethics Equivalency, built around a voluntary agreement signed by countries, funding agencies, philanthropies, and healthcare, patient advocacy, and research organizations, advocates structural global governance reform. The Safe Harbor confronts the challenges we face in bridging 21st century internationally collaborative data-driven genomics research with an increasingly anachronistic ethics review system. Now is the time for the international community to come together and act with a unified voice.

## Box 1. Threshold criteria to engage IFER ethics review

• Human subjects research

- The proposed project must be a systematic investigation designed to develop or contribute to generalizable knowledge and must involve data obtained through interaction with living or deceased natural persons.

• Scientific validity

- The research project’s design and aims must be well-founded, conform to generally accepted scientific principles, and be based on comprehensive knowledge of the scientific literature, as determined by funding or granting agencies.

• Consortia of international scope

- The research project must be managed by a consortium or similar association comprised of member researchers or organizations from more than two countries. Specifically, the multinational scope of the project must involve researchers and data transfer from more than two countries.

• Genomics and health data-focused

- The research project must integrate genomics data into the study design, but may also involve other health-related data such as medical records, stored biological samples, biomarkers, phenotypic, environmental, epidemiological, and clinical trial data.

• Non-interventional

- The research project must not involve direct physical interventions in a person, such as clinical trials involving pharmacologic agents or devices.

## Box 2. Ten guiding principles of a Safe Harbor Framework for International Ethics Equivalency

• Respect for persons

- Research participants should be treated with dignity and integrity. They should be respected both as beings who are capable of exercising decisions, and also as members in communities who make choices in the context of their relationships.

• Beneficence

- Researchers must have the welfare of research participants as a primary goal, particularly those who are vulnerable.

• Justice

- The benefits and burdens of the research project should be distributed equitably among all groups in society.

• Social and scientific value

- Research projects must be designed to yield important information and new knowledge that has a positive impact for science and society.

• Proportionality

- Ethics review and oversight must be commensurate with the risks to and benefits for research participants.

• Procedural fairness

- The process for ethics review of research projects must be conducted efficiently and consistently in accordance with principles of procedural fairness.

• Transparency

- IFER-approved research projects must be publicly disclosed on the IFER website. The quality and type of disclosure should be current and consistent for ease of reference and searchability.

• Security

- When reasonable, and whenever possible, state-of-the-art measures must be employed to minimize the risk of research projects’ data becoming lost, misused, or unjustifiably altered or destroyed.

• Data integrity and quality

- The data being collected, used, and transferred must be relevant to the research project’s purpose(s). Data must be reliable, accurate, complete, and current. If samples are used in a research project, they must be collected, stored, and processed in a way that preserves their long-term stability, searchability, and integrity.

• Accountability

- Research projects and their principal investigators must be willing to be audited at any time and benchmarked to established standards and metrics of ethics protection. NCO screening determinations may also be periodically audited to ensure international consistency and avoid adverse 'forum shopping’ by principal investigators (PIs).

## Box 3. List of standards to satisfy a Safe Harbor Framework for International Ethics Equivalency

Self-assessment, registration, and compliance

- The research purpose must be legitimate: researchers must intend to extend public knowledge through a disciplined inquiry or systematic investigation that is not in contravention of any applicable laws or fundamental human rights.

- All researchers and staff who are directly or indirectly involved in the research project must agree to not use personal data of research participants in any way that deviates from the research plan, and must not share such data with third parties unless obligated by law.

- All research staff who directly handle personal data must certify that they are trained in security and privacy compliance, as determined by the jurisdiction in which they are situated.

- Researchers are responsible for ensuring that all downstream users of data used in the project are in compliance with data security controls and ethics guidelines (including IFER’s policies and standards) and laws in the jurisdiction(s) hosting the data or research team.

- All research projects that share data and/or samples with downstream users must use a simplified Access Agreement to govern the responsible use of those data and/or samples and set out the enforceable rights and obligations of all parties [52].

Dispute resolution and enforcement

- Research projects must adhere to IFER’s dispute resolution system to investigate and resolve complaints and procedures for verifying international and national compliance, in coordination with NCOs. Failure to comply with the Safe Harbor can lead to sanction by IFER, NCOs, and/or governmental bodies.

- Research projects must be subject to ongoing assessment by IFER, with written attestation by the PI(s) and persons with requisite signing authority to affirm compliance with the periodic assessment and that the research project remains in accordance with the Safe Harbor.

## Abbreviations

BBMRI: Biobanking and Biomolecular Resources Research Infrastructure; EBI: European Bioinformatics Institute; H3Africa: Human Heredity and Health in Africa Initiative; HMP: Human Microbiome Project; ICH: International Conference on Harmonisation of Technical Requirements for Registration of Pharmaceuticals for Human Use; IFER: International Federation for Ethics Review; IRB: Institutional review board; NCO: National coordinating office; PI: Principal investigator; REC: Research ethics committee.

## Competing interests

The authors declare that they have no competing interests.

## Authors’ contributions

All authors (Dove, Knoppers, Zawati) contributed equally to the preparation of this article and have read and approved the final manuscript for publication.
